# Exploring the role of lexis and grammar for the stable identification of register in an unrestricted corpus of web documents

**DOI:** 10.1007/s10579-020-09519-z

**Published:** 2021-01-25

**Authors:** Veronika Laippala, Jesse Egbert, Douglas Biber, Aki-Juhani Kyröläinen

**Affiliations:** 1 University of Turku, Turku, Finland; 2 McMaster University & Brock University, Hamilton, ON Canada; 3 Northern Arizona University, Flagstaff, USA

**Keywords:** Web genre identification, Online data, Text classification, Web genres, Online registers, Web-as-corpus, SVM, Discriminative features, Model stability

## Abstract

The Internet offers great possibilities for many scientific disciplines that utilize text data. However, the potential of online data can be limited by the lack of information on the genre or *register* of the documents, as register—whether a text is, e.g., a news article or a recipe—is arguably the most important predictor of linguistic variation (see Biber in Corpus Linguist Linguist Theory 8:9–37, 2012). Despite having received significant attention in recent years, the modeling of online registers has faced a number of challenges, and previous studies have presented contradictory results. In particular, these have concerned (1) the extent to which registers can be automatically identified in a large, unrestricted corpus of web documents and (2) the stability of the models, specifically the kinds of linguistic features that achieve the best performance while reflecting the registers instead of corpus idiosyncrasies. Furthermore, although the linguistic properties of registers vary importantly in a number of ways that may affect their modeling, this variation is often bypassed. In this article, we tackle these issues. We model online registers in the largest available corpus of online registers, the Corpus of Online Registers of English (CORE). Additionally, we evaluate the stability of the models towards corpus idiosyncrasies, analyze the role of different linguistic features in them, and examine how individual registers differ in these two aspects. We show that (1) competitive classification performance on a large-scale, unrestricted corpus can be achieved through a combination of lexico-grammatical features, (2) the inclusion of grammatical information improves the stability of the model, whereas many of the previously best-performing feature sets are less stable, and that (3) registers can be placed in a continuum based on the discriminative importance of lexis and grammar. These register-specific characteristics can explain the variation observed in previous studies concerning the automatic identification of online registers and the importance of different linguistic features for them. Thus, our results offer explanations for the jungle-likeness of online data and provide essential information on online registers for all studies using online data.

## Introduction

The Internet has provided novel possibilities for all scientific disciplines utilizing text data. In particular, the variety of language use and users as well as the extreme sizes of data available online have attracted the attention of natural language processing (NLP), where online data have been used to improve the performance of automatic syntactic analysis (Zeman et al. 2017) and machine translation (Tiedemann et al. 2016; Srivastava et al. 2017), for example. Similarly, in corpus linguistics, online data have been applied, among others, to analyze the new text varieties that have emerged in online settings (Titak and Roberson, 2013; Berber Sardinha 2018) and to examine rare expressions and constructions that are difficult if not impossible to study in traditional language resources (Schäfer 2016; Huumo et al. 2017).

The potential of online data can, however, be limited by the lack of information on the origins of the retrieved documents, specifically, on their *genre* or *register*^1^ (Biber 1988; Biber and Conrad 2009). Registers, such as a user manual, a news article, and a recipe, are text varieties associated with a specific situational context. Register is one of the most important predictors of linguistic variation (Biber 2012). Therefore, register information would be a useful if not essential prerequisite for both the linguistic analysis of online texts and for the use of online data in the development of NLP methods (see, e.g., Giesbrecht and Evert 2009; Webber 2009; Argamon 2019).

However, the modeling of online registers has faced a number of issues, which have challenged both their large-scale linguistic analysis and automatic identification, often referred to as web genre identification (WGI). In particular, the challenges have concerned (1) the extent to which the registers can be automatically identified in an unrestricted corpus based on the open web and (2) the stability of the developed models, that is, to what extent their performance remains stable towards topical, stylistic, or other idiosyncratic corpus characteristics (see Turney 1995 for discussion about stability in machine learning). Furthermore, although descriptive studies have shown that registers differ widely in their lexico-grammatical characteristics (e.g., Biber 2012), these differences are often disregarded in text classification studies on registers. In this article, we focus on these issues. Our research questions are:

1. To what extent can online registers be automatically identified in a large and unrestricted corpus?
2. What kinds of lexico-grammatical features provide the best and most stable results, i.e., what features achieve the best performance while reflecting the registers instead of corpus idiosyncrasies?
3. What are the differences among individual registers with respect to research questions 1 and 2?

By answering these questions, we seek to find answers to the challenges previous studies on online registers have faced. We present our potential solutions to them below.

The first challenge concerns the representativeness of the applied online register corpora and linguistic variation displayed by the Internet. Whereas “traditional” language resources typically represent a pre-determined set of clear-cut register categories, the Internet is much larger and more diverse, including also registers that are less recognized (see Biber and Egbert 2018). Hence, the Internet as a source of data has been described as a jungle in comparison to the properly organized “traditional” language corpora composed of well-known register classes with clear-cut boundaries. These are said to look like well-maintained, organized English gardens (Kilgarrif 2001; Sharoff 2008). Importantly, in WGI, studies have lacked corpora representing this wide range of linguistic variation found online. This has imposed challenges to the generalizability of their outcomes, and many studies have proposed contradictory findings on the extent to which online registers can be identified (Sharoff et al. 2010; Asheghi et al. 2014; Pritsos and Stamatatos 2018 among others). In this study, we model online registers in a massive set of 26 registers and 25,178 documents from the Corpus of Online Registers of English (CORE) (Biber et al. 2015). CORE is currently the largest existing collection of online data with register information. It is based on an unrestricted sample of documents from the open web, without any pre-determined limitations on the included documents or register categories. This ensures that our results are cover the wide range of linguistic variation found on the open Internet. At the same time, naturally, this makes the identification of registers more challenging, as the corpus is not organized into a priori defined, clear-cut classes.

The second challenge in the modeling of online registers concerns the stability of the models. Specifically, Petrenz and Webber (2011) noted that stability “should be added to the evaluation criteria for new approaches to AGC [Automatic Genre Classification].” In previous text classification studies on registers, we can identify three main methods of investigating stability. First, the standard method is to test the performance of the model on another corpus with similar classes. Second, a more qualitative method has been presented by Sharoff et al. (2010) who manually examined the most important features estimated by their model, showing that the features reflected corpus-specific topics rather than generalizable characteristics of a register. Third, Petrenz and Webber (2011) used a dataset that consisted not only of register but also topic information. By varying the distribution of topics in the test data, they demonstrated that their model performances were highly dependent on topics instead of registers.

Our approach to stability builds on these previously applied methods. We start by extending the approach by Petrenz and Webber (2011) by varying the distribution of all the features present in the data, while keeping the distribution of registers constant. In this manner, we can analyze the stability of the model toward all idiosyncratic variation. We introduce this variation by training and testing the model 100 times on different parts of the large CORE The distribution of registers is the same across all the corpus parts, but the distribution of features varies due to topical and stylistic differences between documents. By evaluating the stability of the model performance toward all this variation we can examine to what extent the performance depends on features associated with specific parts of the data instead of the registers.

In addition to the simple stability of the model performance towards changes in the data, an important question in the stable modeling of registers is the relationship between the stability of the model, the model performance and the features selected in the model: what kinds of linguistic features provide the best performance while reflecting the registers in a stable manner, beyond corpus idiosyncrasies? In previous studies, grammatical information has been found to be the most stable toward idiosyncratic variation. However, models trained primarily on grammatical features do not achieve very high accuracies (for results on stability, see Petrenz and Webber 2011; on the use of grammatical features in register identification, see Biber and Egbert 2015). The best-performing models vary across studies, but typically, they are based on lexical information, such as character fourgrams (Sharoff et al. 2010) and word trigrams (Pritsos and Stamatatos 2018). However, as shown by Sharoff et al. (2010) and Petrenz and Webber (2011), these feature sets tend to learn topical aspects associated with the documents rather than generalizable register characteristics. Thus, the question on the best-performing but stable features is still open.

To investigate the kinds of features that provide the highest and most stable performance, we use a linear support vector machine (SVM) in the study. In addition to having achieved high performance in register identification tasks (see Sharoff et al. 2010; Pritsos and Stamatatos 2018) and in document classification in general (see Joachims 1998), an important property of a linear SVM is that it allows us to estimate the contribution of a given feature or feature set to the model (see Guyon et al., 2002; Guyon and Elisseeff 2003). This is essential in our study, as the objective is not only to automatically identify the registers but also to examine the stability and the role of different linguistic features in the model. We present a large scale analysis of register identification based on six feature sets (see Sect. 3.2.1 for details). In addition to experimenting with previously high-performing feature sets, we examine different combinations of lexical and grammatical information. We hypothesize that this combination achieves the high performance previously associated with lexical information while retaining at least some of the stability associated with grammatical features. We start the evaluation of the models by reporting the standard classification metrics. Then, we examine the variance of the model performance as discussed above, in order to analyze to what extent the performance depends on specific corpus parts. As a third step, we evaluate the role of different linguistic feature sets as suggested by Sharoff et al. (2010). First, we analyze the importance of lexical and grammatical features in model performance quantitatively, by increasingly reducing the amount of information available for a given model. If an important source of information, such as grammatical features, is not available, we can expect a drastic drop in the model performance (see Sect. 3.2.3 for discussion). Second, we identify and qualitatively analyze the most important linguistic features—discriminative features—in the models. These allow for a detailed examination of the role of lexical and grammatical information in the model and the evaluation of the extent it targets registers instead of idiosyncratic corpus features.

Finally, a last issue on the modeling of the registers we tackle concerns potential register-specific differences. Typically, studies do not focus on these differences, although descriptive work has shown that the linguistic properties of registers vary extremely. That is, studies have demonstrated that registers differ widely in the extent to which they are well-delimited linguistically and situationally. For example, most texts within the register of Encyclopedia article are highly similar in their linguistic and situational characteristics, in contrast to the extensive variation among texts within the register of News articles (see Biber and Egbert 2018, Chap. 5 and 7; Biber et al. 2020). These differences may affect crucial aspects of the modeling of the registers. Specifically, the extent of inner variation is related to the extent to which registers can be identified, and the varying contribution of grammar can indicate that the discriminative importance of grammar differs between registers. Thus, registers should be considered individually in terms of these aspects.

The article is structured as follows. We start by presenting previous studies on online register identification and stability in Sect. 2. In Sect. 3, we introduce CORE and discuss the methodological steps we took to examine the stability of the models and the importance of different lexico-grammatical feature sets. The results are presented in Sects. 4 and 5. We start in Sect. 4.1 by evaluating the model performance and stability globally, across all registers and feature sets. In Sect. 4.2, we compare the importance of lexis and grammar in the models. In Sect. 5, we move on to more detailed analyses of the models by focusing on register-specific differences. In Sect. 5.1, we present a register-specific evaluation of the best-performing model, and then, in Sects. 5.2 and 5.3, we examine the variation of the discriminative importance of lexis and grammar across registers. Overall, our results demonstrate the importance of grammar in the modeling of registers and highlight the need to consider stability and register-specific differences in their identification. We show that (1) competitive classification performance on a large-scale, unrestricted corpus can be achieved through a combination of lexico-grammatical features (Sect. 4.1), (2) the inclusion of grammatical information improves the stability of the model, whereas many of the previously best-performing feature sets are less stable (Sect. 4.2), and that (3) registers can be placed in a continuum based on the discriminative importance of lexis and grammar (Sect. 5). This reflects how the discriminative importance of topics and specific lexical items varies across registers, and explains why the best-performing feature sets have varied in previous studies—they have focused on different sets of registers with different linguistic characteristics.

## Previous studies on modeling online registers

Registers are text varieties defined by their situational characteristics (see Biber 1988; Biber and Conrad 2009). Registers can be analyzed by their pervasive linguistic characteristics that have a functional basis, i.e., they are functionally related to the situational characteristics and the communicative purposes of the register. For instance, questions can reflect direct interaction between participants of a conversation, which explains their frequency in registers related to conversations. The linguistic characteristics of registers can also be used for their modeling and automatic identification. In this section, we start by introducing previous studies that have focused on the identification of registers and then move on to discussing issues pertaining to the stability of the models.

### Detecting online registers

To date, the majority of studies on the automatic identification of online registers have been based on corpora that present limitations: they tend to be small and topically skewed, or they have been sampled to represent a restricted set of online registers that do not necessarily reflect the full range of linguistic variation found in online data. Consequently, the results do not necessarily generalize to the jungle-like Internet with an extreme range of linguistic variation. For instance, Sharoff et al. (2010) compared various feature sets consisting of part-of-speech information, character n-grams, and lexical information to detect registers in six widely used online register corpora. The sizes of these corpora ranged between 7 and 70 registers and 250–6200 documents. However, even the corpora with a large number of registers did not represent the full range of registers in the Internet. The best accuracies reported by Sharoff et al. (2010) for these corpora were 86% and 97% using character four-grams as binary features. Additional high scores were achieved with fourgrams as non-binary features and bag-of-words. Similarly, Pritsos and Stamatatos (2018) recently achieved an F1-score of 79% based on word trigrams and two of the same corpora that Sharoff et al. (2010) used. For other, earlier WGI studies, see Stamatatos et al. (2000) based on common words, Kanaris and Stamatatos (2007) based on character n-grams, and Santini (2007) based on structural web page information. For a full list of the most important WGI corpora and their sizes, see Biber and Egbert (2015). Despite the high accuracies, further analyses on these WGI studies indicated that the systems lack stability and that the reported results cannot be generalized (see Sharoff et al. 2010; Petrenz and Webber 2011; Sect. 2.2). Similarly, Pritsos and Stamatatos (2018) reported that their best-performing feature sets varied by the used corpora, which suggests that their models may have learnt patterns associated with topics rather than registers.

To enable the development of more stable systems, Asheghi et al. (2016) presented the Leeds Web Genre Corpus, which consists of 15 genres and 3964 documents. The Leeds Corpus was collected by first defining a set of registers (or genres) exclusively used on the Internet and then manually selecting the documents to represent these categories. In Asheghi et al. (2014), the authors presented two sets of experiments using this corpus. In the first one, they did a classification study on the data, achieving a 78.88% accuracy on the plain texts based on character n-grams and an 89.63% accuracy based on a combination of lexical and grammatical features as well as text statistics and boilerplate information, such as headers and links. In the second experiment, Asheghi et al. (2014) exploited the graph structure of the web to increase the accuracy and employed the children of the target web pages as unlabeled training data. Using this semi-supervised graph-based method, their accuracy improved to 90.11% based on bag-of-words. Their study showed that online registers can be identified relatively reliably in a larger and topically diverse corpus. However, because the Leeds Web Genre Corpus is restricted to registers that are used exclusively on the Internet and because the corpus documents are explicitly chosen to represent the registers, the model does not necessarily generalize to the full range of registers and linguistic variation found in the jungle-like Internet.

Currently, the largest collection of online registers is CORE, the one we use in our study (Egbert et al. 2015). CORE is based on an unrestricted sample of the English-speaking open web. Importantly, the documents have not been selected to represent the registers. This means that the corpus represents the full range of linguistic variation also within the register categories. With nearly 50,000 documents, CORE is also substantially larger than the previously applied online corpora. Biber and Egbert (2015) presented the possibility of automatically detecting the CORE registers with stepwise discriminant analysis achieving a precision of 34% and a recall of 40% based on 44 grammatical features. Considering the small number of grammatical features used in their study, the results are promising. However, the performance of the model does leave significant room for improvement and call for the use or more diverse set of features and more flexible models. How well online registers can be automatically identified in the jungle-like, unrestricted Internet is still an open question. Our study provides one answer to this.

### Towards stable identification of registers

In machine learning, stability is used to refer to the repeatability of a learner’s results (Turney 1995). We use it to describe the robustness of a method towards topical or stylistic changes taking place within a corpus or between corpora similar to Petrenz and Webber (2011). A stable system can generalize beyond the training data, and it produces consistent results when given different batches of the same data.

In the context of online register identification, a stable model would be able to identify registers accurately across different corpora or in different parts of one large corpus. This has, however, proven to be a challenge, as noted already in Sect. 2.1. Stability of a system can be evaluated in a number of ways. Sharoff et al. (2010) used variable importance to evaluate stability and whether the most important features in the model targeted registers. They concluded that their model depended on corpus-specific topical features rather than generalizable characteristics reflecting registers. Petrenz and Webber (2011) examined system stability by changing the topic distribution of the test corpus. They compared different feature sets that had been reported to have high performance in previous WGI studies and noted important decreases in the performances. Thus, they concluded that the models were not stable, as they were highly dependent on the idiosyncratic, topical characteristics of the training corpus documents, not the actual registers.

The studies by Sharoff et al. (2010) and Petrenz and Webber (2011) demonstrate the importance of stability in register identification studies. However, the methods they applied have limitations. A simple analysis of the most important model features is informative but relies solely on the qualitative level. The variation of the topic distribution, then, requires a corpus tagged with topic information and assumes that topics are independent of registers. This, however, is not necessarily the case. Theoretically, registers are defined based on their situational characteristics (see Sect. 1; Biber 1988; Biber and Conrad 2009), and not associated with specific topics. Similarly, also Petrenz and Webber (2011) pointed out that an ideal automatic genre classification system “should be stable in the face of changes in topic distribution”. However, Asheghi et al. (2016) noted inevitable correlations between topics and registers such as recipes. Thus, analyzing stability by focusing on topical information can be restrictive. Our approach to stability extends the variation of features from topical ones to a wider range of features, specifically, all the model features. Additionally, similar to Sharoff et al. (2010), we analyze also qualitatively the most important features—discriminative features—of the models. These give information not only about the stability of the models but also about their topicality (see Sect. 3.2.3).

Finally, a stable register identification system would require features that reflect registers beyond topical, stylistic or other idiosyncratic variation attested in a specific corpus. There is, however, no consensus on what these should be. The best-performing feature sets tend to vary across studies (see Sect. 2.1). Typically, the highest scores are achieved by systems based on lexical information that, as we explained, is often related to topics and thus does not necessarily generalize beyond the distribution of the topics in the corpus at hand. Grammatical information typically applied in corpus linguistic register studies (e.g., Biber and Egbert 2015) can be seen as stable towards this topical variation. Also Petrenz and Webber (2011) offered evidence that part-of-speech information provided the most stable results. At the same time, the models trained on grammatical information do not achieve very high accuracies (see Biber and Egbert 2015). Thus, the question of how to use these features to maximize both model performance and stability is still open. Our study analyzes this question from a number of perspectives. In addition to examining the stability by varying the distribution of features, we tackle the importance of different feature sets quantitatively, by proportionally reducing the features used to train the model, and qualitatively, by examining the discriminative features. More information about these methods are given in Sect. 3.2.

## Data, methods, and model fitting

In this section, we discuss the data and methods used in this study. In Sect. 3.1, we start by presenting our corpus, CORE (Biber et al. 2015). In Sect. 3.2, we move on to presenting our methods: SVMs, the different linguistic features included in the modeling, and the steps we take to examine model stability and the importance of the feature sets.

### CORE, the Corpus of Online Registers of English

CORE (Egbert et al. 2015), is currently the largest collection of online documents (*N* = 48,571) with manually annotated register information. The web documents in CORE were selected on the basis of a large number of pseudo-random Google searches. As a result, unlike many other online corpora (see Sect. 2.1), the documents do not represent a limited set of pre-determined registers, but the corpus is representative of the full range of variation in registers and language use that an end user of the internet is likely to encounter when performing Google searches. The registers in CORE represent a hierarchical taxonomy created in a data-driven manner in order to represent the full range of registers found in the corpus. Each document was classified by four annotators, and the final register category of a given document was formed by majority vote. The resulting taxonomy consists of eight general registers that branch into 33 sub-register categories. For a more detailed description of the register taxonomy, the annotation process, and the evaluation of the quality of the annotation, see Biber et al. (2015) and Biber and Egbert (2018).

In this study, we focus on the sub-register level as this makes the comparison to previous studies on online register variation easier. For the sake of simplicity, we refer to these sub-registers as registers. Additionally, we restricted the data to those documents that had a high inter-rater agreement, namely three out of four. This allowed us to focus on the documents that belong clearly to a given category. This yielded the final sub-corpus of 25,176 documents. As CORE is based on an unrestricted sample of the web, the (sub-)register categories are not evenly distributed but can be interpret as reflecting the distribution of registers in the population as a whole, i.e., in the Internet. Table 1 presents the distribution of the (sub-)registers used in this study as well as the average token frequency per document of each (sub-)register.

**Table 1:** Distributional information of the CORE sub-registers used in this study

Register	Documents	Token count (*M*)	Token count (*SD*)	Register	Documents	Token count (*M*)	Token count (*SD*)
Advice	317	1245	2251	Opinion blog	2064	2373	6252
Description of a person	365	1329	2066	Personal blog	1718	1247	3213
Description of a thing	1584	820	2833	Poem	54	844	1506
Description with intent to sell	691	1077	2292	Question/answer	911	803	3749
Discussion forum	1810	1186	2157	Recipe	126	820	643
Encyclopedia article	465	3583	3801	Religious blog/sermon	461	2346	3260
FAQ about information	108	1719	1693	Research article	419	2482	6777
Formal speech	22	4578	7347	Review	1145	1334	3952
Historical article	206	2390	8022	Short story	183	3178	5978
How-to	837	1201	1616	Song lyrics	527	488	1274
Informational blog	337	1788	2692	Sports report	2444	1065	1669
Interview	275	1928	1646	Travel blog	128	1395	2630
News article/news blog	7967	1074	2518	TV scripts	12	3649	2682

For each (sub-)register the average token frequency and standard deviation per document is provided

Table 1 shows the distribution of the sub-register classes in CORE. By far the most frequent is News articles/news blog, which covers nearly 8000 documents, while the least frequent categories are Poem, Formal speech, and TV scripts. While the imbalanced distribution of the registers imposes difficulties for modeling the data, we did not balance them in order to keep the distributions as realistic as possible.

### Methods

In the analysis, we used text classification and linear SVMs (Boser 1992; Vapnik 1998). SVMs have been successfully applied in recent years with state-of-the-art performance in a number of different NLP tasks, including WGI (Petrenz and Webber 2011; Asheghi et al. 2014; Pritsos and Stamatatos 2018; also Rodrigues and Couto 2017). Furthermore, linear SVMs have the advantage that they can be used to estimate the importance of a feature set or a feature to the model performance (see Guyon et al. 2002; Guyon and Elisseeff 2003). This allows us to evaluate the discriminative importance of different linguistic feature sets for registers. Additionally, we can rank the contribution of a particular feature to the model performance and identify the most important ones. We will refer to these as discriminative features and demonstrate that they be used to further analyze the model, its stability and whether it targets registers or corpus idiosyncrasies.

In this section, we start by presenting the feature sets used in this study (Sect. 3.2.1), then move on to introducing the model fitting (Sect. 3.2.2), and finally discuss the steps we take to examine the stability of the models and the role of lexical and grammatical information in them (Sect. 3.2.3).

#### Feature sets

In previous studies, the best results on register identification have been achieved with various versions of word-based features: character fourgrams as binary features (Sharoff et al. 2010), bag-of-words and word trigrams (Pritsos and Stamatatos 2018). While models trained solely on grammatical information utilized in linguistics tend to suffer from poorer performance, they seem to be more stable towards topical corpus characteristics (see Petrenz and Webber 2011). In this study, we compare seven sets of features representing lexical, grammatical as well as character-level document information. The feature sets cover: (1) a pure lexical feature set consisting of words as they occur in a given document, (2) word trigrams, (3) character fourgrams, (4) character fourgrams as binary features, (5) grammatical tags, (6) a *combined* lexico-grammatical feature set consisting of tokens and their associated grammatical tags, and (7) an *independent* lexico-grammatical feature set consisting of tokens and their associated grammatical tags treated as independent features. It is worth pointing out that the difference between the combined lexico-grammatical features and the independent lexico-grammatical features is that in the latter, the token and the grammatical tag are separated as independent features. We illustrate the realization of these feature sets in Table 2 with the example sentence *Fishing is a great way for people to enjoy themselves*. The grammatical information as well as the tokenization were generated with the Biber tagger that is widely applied in register studies and reported in Biber et al. (1999). Additionally, during the preprocessing, the texts were lower-cased, but no stopwords or minimum/maximum frequencies were used.

**Table 2:**
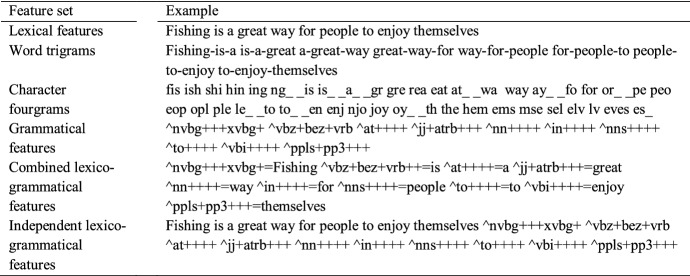
The feature sets compared in the analysis along with examples

To use these features as predictors in in automatic register identification, see Sect. 3.2.2, we calculated their frequency in a given document. Frequently occurring features, such as the words *have* and *and,* are, however, known to carry less information (Manning et al. 2008). Furthermore, absolute frequency of occurrence does not factor in how widely a given feature is used across the documents, and features that are used across multiple documents do not necessarily provide much information in order to differentiate registers. For these reasons, we weighted the frequency of the features using term frequency—inverse document frequency (tf-idf). This is a commonly used weighting scheme in information retrieval (Jones 1972), which assigns a higher weight to features occurring more frequently in a small number of documents and a lower weight to features occurring in a large number of documents.

#### Model fitting and evaluation

The SVMs used in this study were implemented in Python with Scikit learn.^2^ To ensure that the distribution of the registers was as similar as possible between the training and the test sets, the data were split into training (80%) and test (20%) using stratified random sampling. We fitted altogether seven different models to the data to evaluate the performance of the feature sets described in Table 2. Character fourgrams were tested as binary and as non-binary versions. During the model fitting, grid search was used to find the optimal value for two hyperparameters: C associated with the model’s sensitivity to misclassification error and L1/L2 regularization associated with the model’s generalizability (Ng 2004). Regularization allowed us to reduce the dimensionality of the data in a principled manner as high-dimensional data are known to impose difficulties in terms of interpretation, and they can also lead to difficulties in estimating the contribution of a given feature. In the evaluation of the model, we applied three classification metrics to quantify the performance: precision, recall and F1-score (the harmonic mean of precision and recall).

#### Stability and the importance of features and feature sets

To examine the stability of the models, we extended the analysis beyond classification metrics in a number of ways. As a first procedure, we examined the variance of the performance of the models by introducing small changes in training and test data while keeping the distribution of the registers stable (see Efron and Tibshirani 1993 for discussion about sampling procedures). Specifically, we ran the classifier 100 times with the best-performing regularization and C value. In each run, the training and the test data were resampled based on different parts of CORE. Thus, we can quantify to what extent the performance depended on specific parts of the corpus, and evaluate model stability: higher variance in the metrics implied that the performance was sensitive to topical and other idiosyncratic variation in some parts of the corpus, while a lower variance indicated a more stable model (see Petrenz and Webber 2011).

As a second procedure, in order to acquire further information on the contribution of grammar to model performance, we implemented a feature reduction procedure. This procedure follows the idea that only informative features affect model performance, and by varying the informativeness that is available for a given model, we can distinguish between informative and non-informative features (Breiman 2001). Specifically, after training the model, we evaluated its performance on a reduced test set, where a randomly selected set of features was set to zero. This reduction was systematically increased in 10% increments starting from 10% and stopping at 50%. For each 10% increment, the feature reduction was randomized and repeated 100 times to ensure broad coverage of features and documents. The results of this procedure are presented in Sect. 4.2.

As a third and final procedure, we examined the features that were estimated as the most important ones—the discriminative features—in the classification models. During each of the 100 sampling rounds, we recorded 50 of the features with the largest estimated positive coefficient.^3^ By analyzing them, we can qualitatively estimate whether the model targets registers or not (see Sharoff et al. 2010). Furthermore, due to the 100 sampling rounds, we can estimate the selection frequency of the features, that is, how often a given feature was ranked among the top 50 features across the rounds. This offers another, quantifiable perspective to investigate the extent to which a given feature represents a stable property of a specific registers.

## Identifying CORE registers and the stability of the models

In this section, we start presenting the results of the study. First, we examine the performance of the classifiers based on different feature sets (see Sect. 3.2.1) as well as the stability of the models. Then, we analyze the importance of lexical and grammatical information in them.

### Global performance and stability of the models

We experimented with six feature sets (see Sect. 3.2.1). The results of the best-performing models for each of the feature sets are reported in Table 3. The scores represent weighted averages across the 100 iterations and 26 registers.

**Table 3:** Results of the best-fitting SVMs averaged across the 100 sampling iterations and registers for each of the six feature sets and model parameters of regularization (reg) and cost (C)

Feature type	Precision %		Recall %		F1-score %		Model parameters	
	*M*	*SD*	*M*	*SD*	*M*	*SD*	Reg	C
Independent lexico-grammatical	74.48	12.99	75.13	17.40	74.51	15.03	L2	14
Combined lexico-grammatical	73.93	12.76	74.80	18.85	73.88	15.63	L2	20
Lexical	71.84	16.81	70.77	17.29	70.78	16.18	L2	4
Word trigram	70.73	15.28	70.87	22.76	69.01	17.98	L2	5
Binary character fourgram	69.28	17.50	69.04	17.58	69.01	17.27	L2	7
Character fourgram	69.17	17.48	68.98	17.66	68.92	17.29	L2	9
Grammatical	64.34	18.76	59.14	18.32	59.92	17.22	L1	16

First of all, the results in Table 3 demonstrated that both lexical and grammatical features were required to achieve the best results: both the combined lexico-grammatical and the independent lexico-grammatical feature sets outperformed the third best classifier, that is, the lexical one (weighted Welch *t* tests *t(*4832.46) = −28.56, *p* = 0.00 and *t*(4380.65) = −47.49, *p* < 0.001). This demonstrates that adding grammatical information improved the identification results. The difference in the model performance (F1-score) with the combined and the independent lexico-grammatical feature sets was not statistically significant (*t*(5190.06) = 1.5, *p* = 0.13). However, the independent model is the preferred one because it is simpler in terms of number of features.

The results between the lexical classifier and the other word-based classifiers, that is, the word trigram and the two character fourgram classifiers, were very similar. Considering that the previously reported best results were achieved with these feature sets (Sharoff et al. 2010; Pritsos and Stamatatos 2018), their high performances in our experiments is not surprising. Similarly, as expected, the performance of the grammatical classifier was substantially lower than that of the others. Interestingly, however, the grammatical classifier was also the only one where recall was clearly lower than precision. This suggests that all necessary information needed to model registers reliably could not be covered by grammar. Lexical information, in contrast, seems to provide more flexibility and opportunities for a model to learn structures associated with a given register.

With the overall F1-score of 74.5%, the results demonstrated that linguistic features allow us to identify online registers relatively well even when the data are based on the data-driven CORE and the distribution of registers is highly unbalanced. In comparison to previous studies, our best model clearly outperforms the precision of 32.9% and recall of 41.5% reported by Biber and Egbert (2015) on effectively the same data (see Sect. 2.1. About model performances in previous studies on various data sets).

In addition to showing that the combination of lexical and grammatical information provides the best modeling results, Table 3 brings forth the differences in the stability between different models. These are reflected by the standard deviations of the classification metrics, as the standard deviation reflects the variation of the model performances across different parts of the corpus (see Sect. 3.2). Specifically, the models trained on the independent lexico-grammatical features and the combined lexico-grammatical feature sets displayed both lower degrees of variance and thus higher degrees of stability than the lexical model: F1-score (*F*_2599,2599_ = 0.88, *p* < 0.001), precision (*F*_2599,2599_ = 1.09, *p* = 0.02) and recall (*F*_2599,2599_ = 0.878, *p* < 0.001). This similar pattern of increased stability was repeated when comparing the *SD* of the independent lexico-grammatical model to the *SD* of the trigram model: F1 score (*F*_2599,2599_ = 1.66, *p* < 0.001), precision (*F*_2599,2599_ = 1.96, *p* < 0.001) and recall (*F*_2599,2599_ = 1.96, *p* < 0.001).

Thus, to sum up the results of the classification experiments, our analysis shows that the combination of lexical and grammatical information provides the highest performance and that when combined to lexical information, grammatical information also improves the stability of the model. In the next section, we move on to further analyze the importance of grammar by comparing the contribution of lexis and grammar in the models and their performances.

### Global contribution of lexis and grammar

**Fig. 1:**
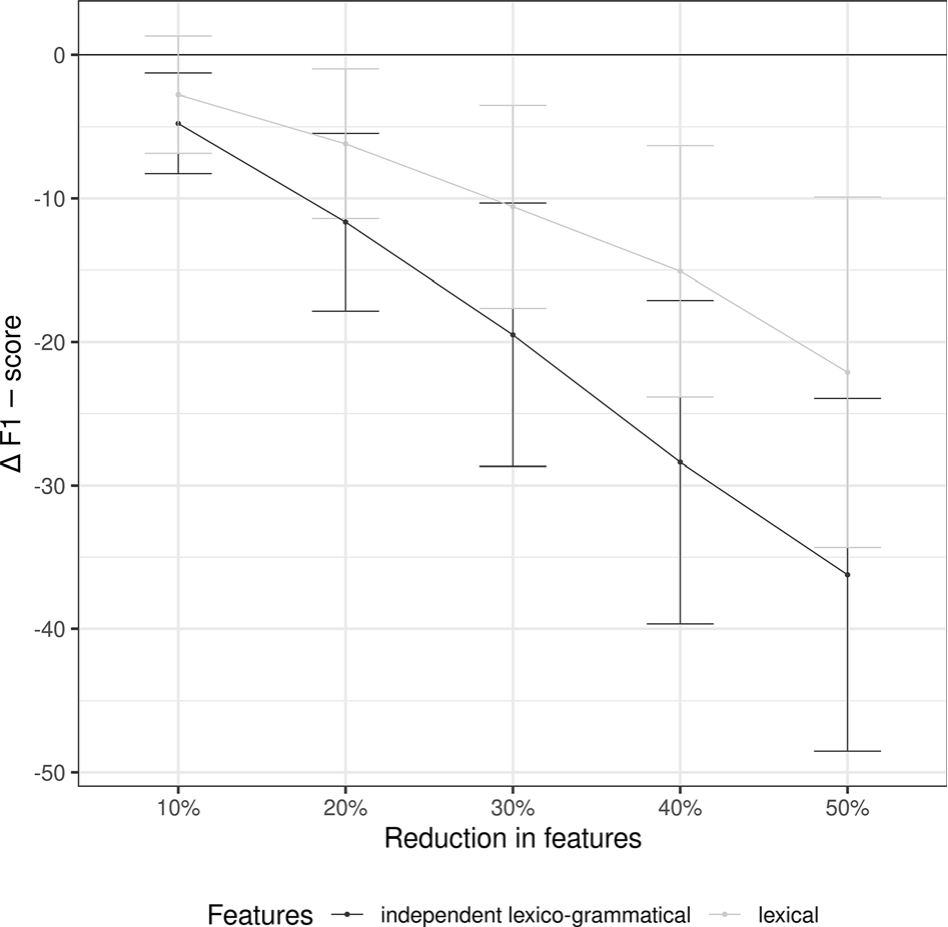
Relative performance of independent lexico-grammatical and lexical models measured as Δ F1-score when the contribution of a proportion of features was set to zero

The contribution of grammatical information shows also when the performances of the independent lexico-grammatical classifier and the lexical classifier were compared against reduced test sets where a proportion of randomly selected features was set to zero (see Sect. 3.2.3 for details of the method). The results of the experiment for the two classifiers are presented in Fig. 1. The differences between the performances are measured as Δ F1-score between a full test set and a reduced one. The reduction was systematically increased stepwise in 10% increments.

The differences in the performances of the two classifiers are clear. The performance of the independent lexico-grammatical classifier reacts to the reduced sets of features by decreasing much more than the lexical classifier does. Already at the reduction of 20% of the features, the difference between the F-scores is more than 5%, and at 50%, the difference is approximately 15%. This shows how the loss of important information—grammar in this case—affects the identification of registers. Importantly, there is also an increase in variability, reflected in the width of the confidence intervals. This further supports our finding.

The importance of grammatical information for registers shows also in the discriminative features of the register classes. In Table 4, we present the discriminative features estimated for News along with their selection frequency, that is, how often a particular feature was selected among the most important features across the sampling iterations during the model fitting procedure (see Sect. 3.2.3). The grammatical features include public verbs and reporting verbs (red text in the table), prepositions (purple text), past tense verbs (highlighted in yellow), perfect aspect (green text) and that-deletions (highlighted in purple). The lexical features, in turn, are related to news, politics, and places (highlighted in green), as well as features related to interviews or citations (highlighted in red). Many of these features are similar to those that have previously been associated with news (see Biber and Egbert 2018, pp. 83–84). Thus, the table that the model targets registers relatively well.

**Table 4:**
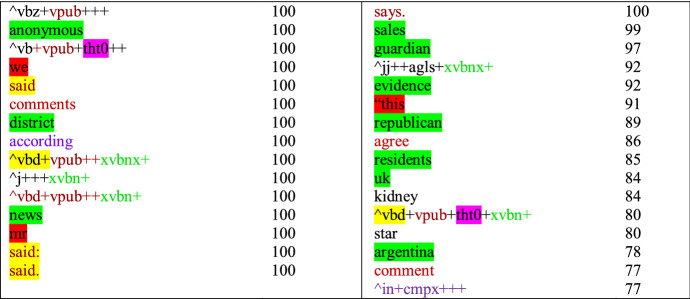
Discriminative features of News along with their selection frequency estimated with the best-fitting SVM

Public verbs and reporting verbs → red text, vpub; Prepositions → purple text, in+cmpx; Perfect aspect → green text, xvbn, xvbnx; That-deletions → highlighted in purple, tht0; Past tense → highlighted in yellow, vbd

The selection frequencies following the discriminative features presented in Table 4 indicated that many of them were selected consistently across the sampling 100 iterations. Their analysis supports the importance of grammatical information and its contribution to the stability of the classifier. Many of the features selected during each of the 100 sampling rounds are grammatical tags. Additionally, the lexical features selected consistently 100 times denote functional elements, such as past tense and verbs included in the public verb category. Only the lexical features selected less consistently refer to topical items that would not seem to generalize to news in general. For instance, *uk* selected 84 times and *argentina* selected 78 times are associated with news article topics instead of the register.

## Register-specific differences in the modeling

Globally, we have presented evidence that in our study setting, online registers are the best modeled with a combination of lexical and grammatical information. Furthermore, grammatical information, especially lexico-grammatical information when defined as independent features, improves the stability of the model as demonstrated in Sect. 4.1. However, the global model performance or stability does not inform us about potential register-specific differences. As language is known to display variation, even extreme, across registers (Biber 2012), such differences can have important effects on the modeling results. In this section, we focus on these. In Sect. 5.1, we present register-specific variation of the identification rates based on the globally best-performing feature sets. In Sect. 5.2, we investigate how the best-performing feature sets, specifically the lexico-grammatical and the lexical, varied across registers. Finally, in Sect. 5.3, we extend the analysis to discriminative features in order to further analyze the models and the importance of grammar in them. Together these aspects bring forth the systematic variation associated with registers and their modeling.

### Model performance by register class

**Fig. 2:**
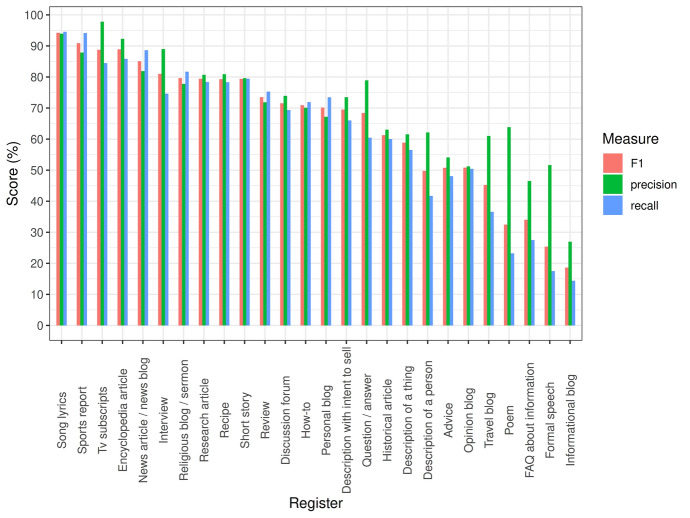
Register-specific performances estimated with the best fitting SVM based on the independent lexico-grammatical features

The best model performance was achieved using the combination of lexical and grammatical information with a 74.5% F1-score. Figure 2 below presents the model performance separately for each register.

In Fig. 2, the results demonstrated important local variation across the registers. The best performances were obtained for Song lyrics, Sports report, TV scripts, Encyclopedia article, and News article/news blog, for which the F1-score was 85% or above. Also, for Interview, Research article, Religious sermon, Recipe, and Short story, the F1-score was nearly 80%. On the lower end, Advice, Description of a person, Information blog, Opinion blog, and Travel blog received an F1-score of 50% or less. The different proportions of the registers in the training data affects undoubtedly the results. In particular, the larger proportion of News afforded more learning opportunities but can also bias the classifier to select it. At the same time, some of the smaller registers, such as Interview, were detected relatively well in comparison to other proportionally larger ones, such as Opinion blog. Consequently, the results also reflect variation in how difficult the registers are to detect.

The fact that some registers might be more difficult to detect than others is supported by the linguistic analysis by Biber and Egbert (2018, pp. 83, 118). They revealed important inner variation in the linguistic characteristics of Opinion blog, Advice, and Personal blog. In our experiments, these registers have also received relatively low scores considering their size. Thus, it would seem that these properties correlate.

### Best-performing model by register class

**Fig. 3:**
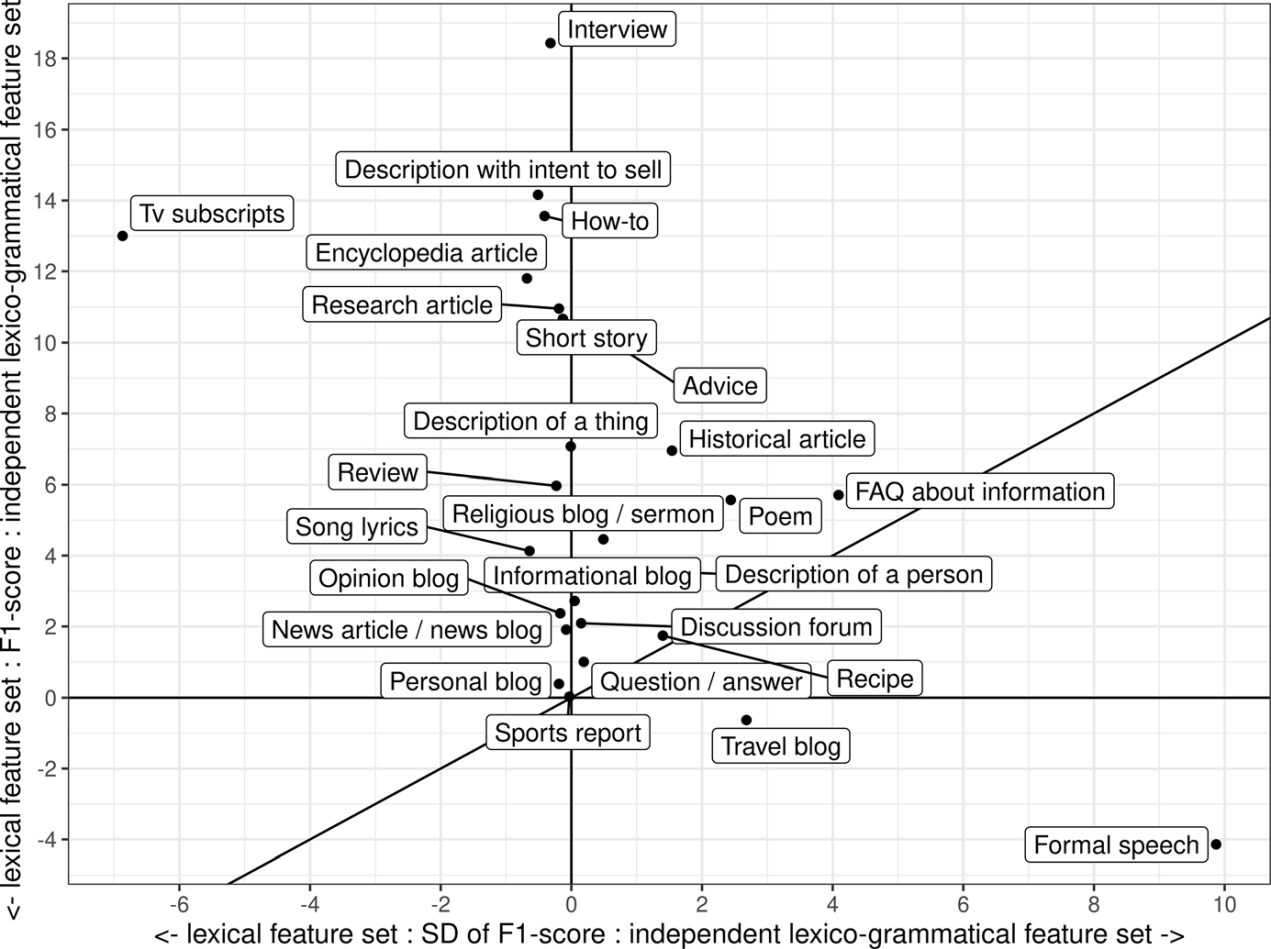
Lexico-grammatical continuum of online registers based on relative differences in the F1-scores and *SD* of F1-score

In Sect. 4, we demonstrated the global importance of grammar in the identification of registers. However, the descriptive results by Biber and Egbert (2018) suggest that the importance of grammar varies across registers. In the following, we examine the best-performing models by register to know if this variation shows in the modeling results. We started by comparing the independent lexico-grammatical and lexical models by register class. We calculated the Δ F1 by subtracting the F1-score estimated with the lexical model from the F1-score of the independent lexico-grammatical model for each register. On this scale, positive values indicate that the model trained on the independent lexico-grammatical information performed better than the lexical one and negative ones signal the reverse situation. Similarly, we also calculated the Δ *SD* score based on the *SD* of the F1-scores for each register. This Δ *SD* score also has the same interpretation as the Δ F1: positive values indicate smaller *SD*s for the lexico-grammatical classifier. This allows us to place individual registers in a continuum according to the discriminative importance of grammar, lexis and stability, and is visualized in Fig. 3.^4^

The Δ scores clearly demonstrated substantial register-specific variation in the best-performing model. On one end, we have Interview, Description with intent to sell, TV script, How-to, Encyclopedia article, Research article, Short story, and Advice. For these, the lexico-grammatical classifier outperformed the lexical classifier by 10% or more. Also Review, Description of a person, Song lyrics, Historical article, Description of a thing, FAQ about information, Opinion blog and Religious blog/sermon benefit from the grammatical information (see the “Appendix” for tests of statistical significance). On the other end, we have Formal speech and Travel blog, for which the lexical classifier scores are slightly higher than those of the lexico-grammatical classifier. In the center, we have registers with similar scores for the lexical and the lexico-grammatical classifiers: Sports report, Personal blog, News article, Question/answer, Recipe, Poem, Discussion forum, and Informational blog. For these, the inclusion of the grammatical information had very little or no improvement to the performance of the model. Finally, also the *SD* varied between the lexical and the lexico-grammatical classifiers. Whereas the *SD* of the lexico-grammatical classifier was lower for most of the registers placed on the right side of the figure, for TV subscripts, the *SD* of the lexical classifier was lower. This can reflect a characteristic of this register, which can include, for instance, repetitive lexical items that cannot be captures by grammar.

Thus, the comparison of F1-scores shows that whether or not the lexico-grammatical classifier outperforms the lexical one depends on the register. This suggests that the discriminative importance of grammar varies across registers. However, a simple comparison of classifier scores may be misleading because lexical features can account for grammatical patterns, too. For instance, although the perfect aspect is the most directly expressed by a grammatical tag, a lexical or a lexico-grammatical classifier can embed the same information by simply listing all verbs in the perfect aspect. Therefore, to provide further evidence for the discriminative importance of grammar, we fitted a new lexical model to the data by reducing the number of lexical features to 4400 corresponding to the number of features used to train the grammatical classifier. This *reduced lexical classifier* achieved a 69.5% precision, a 66.5% recall, and an F1-score of 67.0%.

**Fig. 4:**
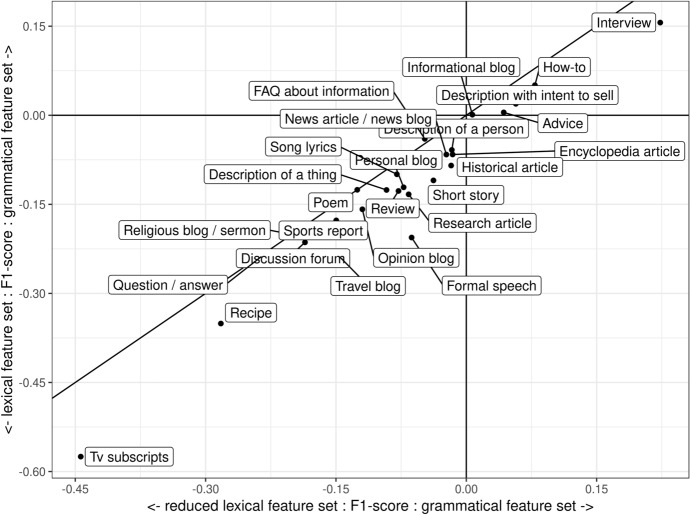
Relative differences in the F1-scores between the lexical classifier, the reduced lexical classifier and the grammatical classifier

We calculated Δ F1 scores for the following model pairs: (1) lexical and grammatical and (2) reduced lexical and grammatical. These relative scores are visualized in Fig. 4. The difference between the lexical and the grammatical feature sets is given on the Y-axis, whereas the difference between the reduced lexical and the grammatical feature sets is given on the X-axis. Positive values on both scales indicate that the SVMs trained on the grammatical feature set outperformed the lexical ones. In other words, the registers that were estimated to be impacted the most by the relative contribution of the grammatical information are located in the top right corner. In contrast, the registers impacted the most by lexical information are located in the bottom left. The identity line separates registers impacted by the reduction of the lexical features, that is, differences in the scores between the reduced lexical classifier and the lexical one.

Figure 4 supports the results presented in Fig. 3 and gives further evidence on the importance of grammar for some registers. The grammatical classifier outperformed the reduced lexical one for Interview (Welch t test, *t(*191.78) = −29.45, *p* = 0.00), Description with intent to sell (*t(*192.01) = −4.16, *p* < 0.001) and How-to (*t*(197.93) = −12.64, *p* < 0.001). For Advice and Informational blog, the difference between the lexical and grammatical model was not statistically significant (*t(*187.41) = −0.93, *p* = 0.35; (*t*(187.41) = −0.93, *p *= 0.35)). All registers except for Poem and FAQ about information were negatively affected by the reduced set of lexical features. In other words, for all but these two, the relative difference between the lexical and grammatical feature sets became smaller.

### Confirming the discriminative importance of grammar by the analysis of discriminative features

In the previous section, the analysis of the model performances suggested that the discriminative importance of grammar varies across registers. In this section, we continue this discussion by analyzing the models qualitatively through discriminative features. We present examples from three registers: Interview, for which lexico-grammatical information displayed the highest performance, and Travel blog and Discussion forum, for which lexical information alone achieved high performance.

#### Registers with a high-performing lexico-grammatical model

In Sect. 5.2, we showed that the contribution of grammatical information to the classifier performance was the highest for Interview. Table 5 presents the discriminative features estimated by the lexical classifier for this register. Of the 14 discriminative features selected during all the sampling iterations, most describe functional patterns.

**Table 5:**
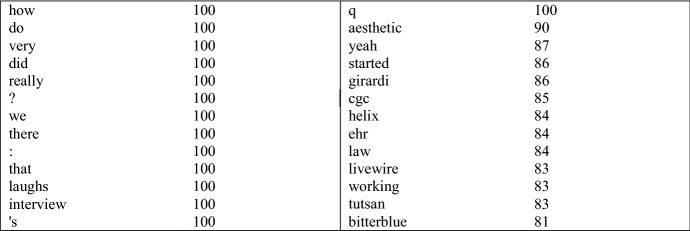
The discriminative features for the interview class, estimated by the lexical classifier

Specifically, the discriminative features in Table 5 are related to interactive discourse that is typical of interviews (see Biber and Egbert 2018, pp. 192–194). The interrogative adverb *how* and the punctuation marks ? and : are related to questions, similar to the abbreviation *q,* which is used in the documents to denote questions asked by the interviewer. *Do* and *did* are auxiliaries that are also often used in questions, whereas the first person pronoun *we* is typically used by the interviewee. *Really* and *yeah* can be used as discourse particles that guide the interaction. For comparison, Table 6 below presents the discriminative features estimated by the grammatical classifier for the same register.

**Table 6:**
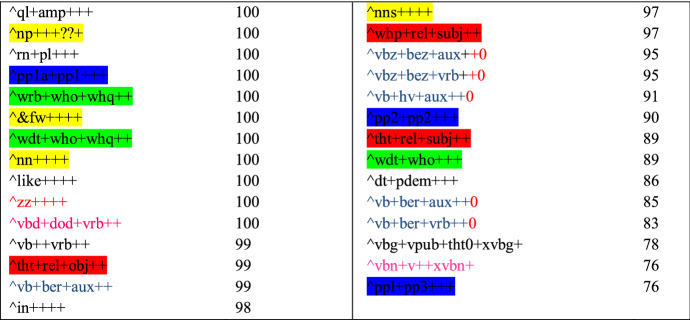
Discriminative features for Interview, estimated by the grammatical classifier

Nouns and foreign words → highlighted in yellow, np, nn, &fw, nns; Interrogative determiners and adverbs → highlighted in green, wrb+who+whq, wdt+who+whq, wdt+who; Relative clauses → highlighted in red, tht+rel+obj, whp+rel+subj, tht+rel+subj; Personal pronouns → highlighted in blue, pp1a+pp1, pp2+pp2, pp1+pp3; Contractions → red text, zz, 0; Auxiliaries → blue text, vb+ber+aux, vbz+bez+aux, vbz+bez+vrb, vb+hv+aux, vb+ber+vrb; Past tense → pink text, vbd+dod+vrb, vbn+v++xvbn; Amplifiers → ql+amp; Adverbs → rn+pl; Determiner/demonstrative pronouns → dt+pdem; Present progressive verb, public verb, that-deletion,-ing form → vbg+vpub+tht0+xvbg

Many of the grammatical functions listed directly in Table 6 are reflected by words in Table 5: auxiliaries, interrogative pronouns and adverbs, past tense, personal pronouns, and the amplifier *very.* Additionally, *that* in Table 5 can correspond to relative clauses and determiner/demonstrative pronouns in Table, and many of the topical nouns in Table 6 would get tagged as nouns. It would thus seem that both lists of discriminative features denote similar, functional constructions that are the most directly reflected by grammar. This confirms the quantitative results on the importance of grammar in the modeling of this register, presented in Sect. 5.2. Additionally, on a more general level, this suggests that the two approaches we have used in this study—evaluation of the classification metrics and of the discriminative features—point to similar findings. Thus, while we cannot examine the discriminative features of all the registers, we can assume that grammatical information is important in the modeling of all the registers for which the classification metrics showed that grammatical information carried clear improvements to the results. In addition to Interview, these registers include Description with intent to sell, TV subscripts, How-to, Encyclopedia article, Research article, Short story, Advice, Review, Description of a person, Song lyrics, Historical article, Description of a thing, FAQ about information, Opinion blog and Religious blog/sermon (see Fig. 3 and “Appendix”).

#### Registers with a high-performing lexical model

In addition to proving the discriminative importance of grammatical information for some registers, the results presented in Sect. 5.2 showed that for some registers, lexical information was sufficient for achieving high performance. For Formal speech and Travel blog, the lexical classifier achieved the best results. Additionally, the model performances by the lexico-grammatical and lexical classifiers were very similar for Sports report, Personal blog, News article, Question/answer, Recipe, Poem, Discussion forum, and Informational blog.

Table 7 below illustrates the discriminative features for Travel blog, as estimated by the lexical classifier that achieved the highest performance for this register. The most frequently selected features denote general vocabulary related to traveling, for example, *visit*, *travel*, *trip*, and *locals*. These are at the same time specific to traveling but also very broad so that they can be associated with a large number of travel blog posts. The only functional patterns among the discriminative features are the pronoun *we* and the past tense in *visited*. Additionally, the features include some more specific topics related to traveling and multiple place names referring to common travel destinations. All in all, these features confirm the model performance presented in Sect. 5.2: lexical, in particular topical information, carries the highest discriminative importance for this register.

**Table 7:**
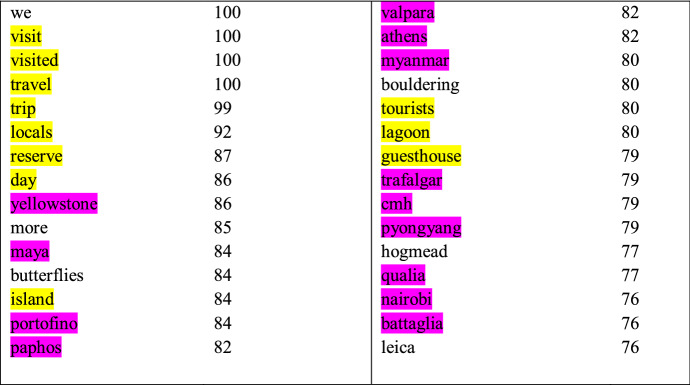
Discriminative features estimated by the lexical classifier for Travel blog

Features denoting general vocabulary related to traveling are highlighted in yellow and common travel destinations are in purple

Many of the registers that were associated with high model performance by the lexical classifier appear to be focused around a specific topic. It is logical that the lexical classifier achieved good performance as these topics can be denoted directly with lexical items. However, the lexical classifier also performed well for other registers without particular inherent topics. These included Discussion forum, Personal blog, Opinion blog, and Question/answer. To illustrate this possibility, the discriminative features for Discussion forum are given in Table 8.

**Table 8:**
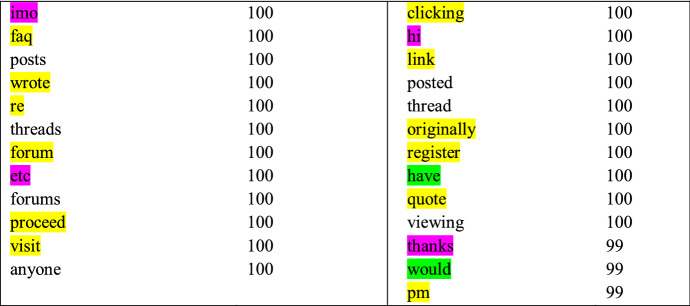
Discriminative features estimated by the lexical classifier for Discussion forum

The discriminative features in Table 8 include some terms that are used in the documents but also in the discussion forum template (highlighted in yellow), abbreviations and interactive patters (in purple), two functional words (in green), and a number of other words related to forum discussions (the rest). Although the large majority of the words do not necessarily reflect a specific topical pattern, they are all tightly related to discussion forums. Moreover, many of them, such as *posts* and *posted*, have very specific, fixed uses and are not easily replaceable by other functionally similar patterns. This explains the success of the lexical classifier, as grammar cannot denote these words directly, and confirms the discriminative importance of lexis for this register.

## Discussion and conclusion

The goal of this study was to model online registers by focusing on two issues that had been largely disregarded in previous research: the extent to which the registers can be identified in corpora representing the full range of linguistic variation found on the open web, and the stability of the developed models. Furthermore, we aimed at paying particular attention to register-specific differences in their modeling. Although previous descriptive studies had shown that registers differ in many aspects that can affect their modeling, these differences are often bypassed in studies aiming at automatically identifying them. Our research questions were (1) To what extent can online registers be automatically identified in a large and representative corpus (2) What kinds of linguistic features provide the best and most stable results, i.e., what features achieve the best performance while reflecting the registers instead of corpus idiosyncrasies? (3) What are the differences among individual registers with respect to research questions 1 and 2?

To examine these questions, we used as a corpus CORE, which is substantially larger than the previously applied online corpora and is based on an unrestricted sample of the English web. The fact that the CORE documents are not explicitly chosen to represent the registers improves the applicability of our results. We first ran register identification experiments on CORE with six feature sets. These included both lexical features based on which previous studies had reported the highest performance (Sharoff et al. 2010; Pritsos and Stamatatos 2018), as well as grammatical features that have been demonstrated to remain the most stable towards corpus idiosyncrasies (Petrenz and Webber 2011). Furthermore, we experimented with combinations of these two feature sets, with the hypothesis that the combinations would combine high performance with stability.

Then, to acquire detailed information on the stability of the models and on the role different features have in them, we extended the standard register identification task in a number of ways. First, we examined the variance of the classification metrics when faced with small changes in the data. Specifically, we trained and tested the models 100 times always shuffling and redividing the data to train and test sets between the iterations. This provided information on how stable the models were towards variation that tends to take place across documents belonging to the same register. Whereas previous studies had focused on performance variance against topical changes in the data (see Petrenz and Webber 2011), we analyzed the variance against changes in all the features. This ensured that we did not restrict the analysis on topical aspects of stability. Second, we evaluated the importance of lexical and grammatical information in the models by extending the approach previously applied by Sharoff et al. (2010). While they analyzed the role of different features in the model qualitatively, by examining the most important ones in it, we evaluated their importance both quantitatively and qualitatively. The quantitative evaluation was done by comparing model performances against reduced test sets, where the amount of information given to the model was proportionally reduced. On the qualitative level, similar to Sharoff et al. (2010) we analyzed discriminative features, that is, the features estimated as the most important in the model. Finally, we compared the results on model performance, stability and the role of different linguistic features across the 26 CORE registers. Thus, the results presented here are based on a large and representative corpus and a detailed analysis of the model stability including both of quantitative and qualitative evaluations.

### Can the jungle of online registers be tamed if not conquered?

Our study revealed three major aspects of online registers and their modeling. First of all, in Sect. 4.1, the results showed that online registers can be identified relatively reliably even in a corpus that is compiled in an unrestricted manner and includes registers that are not the clear-cut stereotypical categories included in many previous studies. Our best performing model was trained on lexico-grammatical information and achieved an average F1-score of 74.51% across the 26 registers in CORE. Considering the challenges previous studies have faced in the automatic identification of registers, this is very encouraging for the future of online register identification. Furthermore, this shows that the jungle of online registers can be tamed if not conquered.

Second, the results demonstrated the importance of grammar in the modeling of registers. Our best performance in Sect. 4.1 was achieved with a model based on both lexical and grammatical information. The differences in the F1-scores of the best-performing model and the models based on lexical information alone (bag-of-words, character fourgrams, word trigrams) ranged between 3.73% and 5.59%. Combining lexical and grammatical information in the model increased also the stability of the model: the models trained on both lexical and grammatical information were more stable towards changes in the data than the models trained on lexical information only. Similarly, the findings in Sect. 4.2 supported the importance of grammar; the loss of grammatical information affected the model performance more dramatically than the loss of lexical information alone, and also the qualitative analysis of discriminative features demonstrated the importance of grammatical features in the models. Thus, our hypothesis on the advantages of combining grammar and lexis is confirmed.

Third, the results highlighted register-specific differences in their modeling. In Sect. 5.1, we showed how the identification performances varied across registers. The highest F1-scores, 85% or more, were achieved for Encyclopedia article, News, Song lyrics, and Sports report, whereas for Advice, Description of a person, Information blog, Opinion blog, and Travel blog, the F1-scores were 50% or lower, with the remaining registers falling between these two extremes. Although the variation is partially resulting from the uneven class distribution in the data, the differences reflect also register-specific variation in how difficult the classes are to identify. For instance, Biber and Egbert (2018, pp. 83, 118) showed that Opinion blog, Advice, and Personal blog display important inner variation. This can also explain their lower identification rates. From a theoretical perspective as well, this variation is motivated considering the differences in the situational characteristics of the registers. Many of the registers that have high identification rates despite representing a small proportion of the data, such as Encyclopedia article, are typically associated with specific situations of use entailing also better defined functional possibilities and linguistic characteristics. In contrast, many registers with lower identification rates, such as Information blog, can be associated with less strictly defined situations and a broader range of functional and linguistic possibilities (see Biber et al. 2020).

In addition to showing register-specific variation in the extent to which they can be identified, our analysis in Sect. 5.2 demonstrated that registers can be placed in a continuum according to the discriminative importance of lexis and grammar. A number of registers were the best identified with the combination of lexical and grammatical information: Interview, Description with intent to sell, TV script, How-to, Encyclopedia article, Research article, Short story, Advice, Review, Description of a person, Song lyrics, Historical article, Description of a thing, FAQ about information, and Religious blog/sermon. For Interview, Description with intent to sell and How-to, the importance of grammar was particularly high as the grammatical classifier outperformed the reduced lexical classifier based on a similar number of features. On the other end, for Formal speech and Travel blog, the lexical classifier outperformed the lexico-grammatical ones, and for Sports report, Opinion blog, Personal blog, News article, Question/answer, Recipe, Poem, Discussion forum, and Informational blog, the performances of the lexical and the lexico-grammatical classifiers were very similar.

The analysis of the discriminative features in Sect. 5.3 gave further evidence on this continuum by showing that the kinds of linguistic features targeted by the models vary across the classes. The most important features for registers for which grammatical information carried discriminative importance were functional patterns that are the best reflected by grammar, whereas the most important features for other registers were the best described by lexical information. These included both topical features associated with, e.g., traveling, and specific lexical items that could not be captured with grammatical tags. Although a detailed analysis of all the registers is out of the possibilities of the current study, this finding does shed light also on the relationship between topics and registers discussed in many register identification studies (e.g., Asheghi et al. 2014; see also Haider and Palmer 2017). Specifically, our analysis suggested that topics are relevant for some registers, such as Travel blog, for which lexical information has high predictive importance, while for others, such as Interview, this is not the case.

### Implications and future directions

To sum up, our study highlights the importance of stability and register-specific differences in their modeling. As already noted by Petrenz and Webber (2011), stability should be considered carefully in text classification. In our analysis, many of the feature sets that were previously reported to achieve the best results, such as word trigrams and character fourgrams, displayed lower degrees of stability. This naturally weakens their application potentials. Considering register-specific differences, then, our findings can explain the contradictory results presented in previous studies on the extent to which registers can be identified and on the kinds of features that best reflect them—if the compared corpora are not composed of similar registers, they may not be easily comparable. On the other hand, from a corpus compilation perspective, this variation needs to be taken into account as well, in order to present registers in a representative manner.

On a methodological level, our results highlight the importance of analyzing the lexico-grammatical aspects of the models and the registers, and, in our case, the use of an SVM. The application of a more widely applied classification approach—such as standard text classification without analyzing the linguistic basis of the model—would not have revealed these results. Thus, our results also highlight the need to combine NLP and linguistic analysis so as to not only achieve high performance but also to guarantee the validity of the results.

Finally, this article inspires a number of new directions. One obvious continuation is a more detailed examination of the registers with distinguishing grammatical, topical and other characteristics. Additionally, the analysis should be extended to the CORE registers with lower inter-rater agreement. Similarly, the use of neural methods should be interesting, and would be likely to provide higher identification performance. Furthermore, it would be interesting to compare our results on the contribution of different feature sets to predicting registers to cognitive experiments on how registers or genres are identified by humans. For instance, Clark et al. (2014) have shown that various typographical features, layout and even numbers are important in interpreting the purpose of e-mails. Our future plans also include the development of a WGI system based on the CORE registers and its application to other corpora. This will also require further analyses on the variation of registers and the stability of their characteristics.

## Appendix

Register-specific F1-scores of the SVMs trained four different feature sets. The results are averages across the 100 sampling rounds.

Results of the Welch *t* test on the differences between the average F1-scores of the SVMs trained on the independent lexico-grammatical feature set and the lexical feature set.

**Table:**

Register	Feature set				*t* value	*df*	*p* value
	Independent lexico-grammatical		Lexical				
	*M*	*SD*	*M*	*SD*			
Description of a person	49.78	5.37	46.21	4.26	5.21	188	< 0.001
Advice	46.21	4.26	40.79	4.18	15.54	194	< 0.001
Review	73.52	1.95	67.55	2.18	20.4	196	< 0.001
Encyclopedia article	88.91	2.18	77.11	2.86	32.8	185	< 0.001
Research article	79.46	3.28	68.5	3.47	22.96	197	< 0.001
Short story	79.4	4.51	68.73	4.64	16.48	198	< 0.001
Song lyrics	94.21	1.23	90.08	1.87	18.44	171	< 0.001
TV script	88.77	16.45	75.77	23.32	4.55	178	< 0.001
Historical article	61.27	5.8	54.32	4.26	9.65	182	< 0.001
Description of a thing	58.87	1.98	51.8	1.99	25.22	198	< 0.001
FAQ about information	34.05	10.67	28.34	6.58	4.55	165	< 0.001
How-to	70.92	2.44	57.36	2.85	4.53	198	< 0.001
Opinion blog	57.36	2.85	48.43	2.08	8.41	196	< 0.001
Description with intent to sell	69.49	2.93	55.33	3.44	31.32	193	< 0.001
Interview	81.01	3.75	62.58	4.07	33.33	197	< 0.001

## Footnotes

1 Following the research tradition established, e.g., in Biber (1988) and Biber and Conrad (2009), we use the term “register” instead of “genre.” However, in the context of their automatic detection, the two notions are essentially interchangeable.

2 http://scikit-learn.org/.

3 While it is certainly possible to analyze features with negative coefficients, we chose to concentrate on the positive ones as they are easier to interpret as their presence is positive associated with a particular register..

4 Register-specific scores for the classifiers discussed in this section are presented in the “Appendix”.
